# Strengthening and stretching for rheumatoid arthritis of the hand (SARAH): design of a randomised controlled trial of a hand and upper limb exercise intervention - ISRCTN89936343

**DOI:** 10.1186/1471-2474-13-230

**Published:** 2012-11-24

**Authors:** Jo Adams, Chris Bridle, Sukhdeep Dosanjh, Peter Heine, Sarah E Lamb, Joanne Lord, Christopher McConkey, Vivien Nichols, Francine Toye, Martin R Underwood, Mark A Williams, Esther M Williamson

**Affiliations:** 1 Warwick Clinical Trials Unit, Warwick Medical School, University of Warwick, Coventry CV4 7AL, UK; 2 Faculty of Health Sciences, University of Southampton, Highfield, Southampton, Hampshire, SO17 1BJ, UK; 3 Health Economics Research Group, Brunel University, Uxbridge, Middlesex, UB8 3PH, UK; 4 Nuffield Orthopaedic Centre, Windmill Rd, Headington, Oxford, Oxfordshire, OX3 7HE, UK; 5 Kadoorie Critical Care Research Centre, John Radcliffe Hospital, Oxford OX3 9DU, UK

**Keywords:** Randomised controlled trial, Rheumatoid arthritis, Exercise, Hand, Rehabilitation

## Abstract

**Background:**

Rheumatoid Arthritis (RA) commonly affects the hands and wrists with inflammation, deformity, pain, weakness and restricted mobility leading to reduced function. The effectiveness of exercise for RA hands is uncertain, although evidence from small scale studies is promising. The Strengthening And Stretching for Rheumatoid Arthritis of the Hand (SARAH) trial is a pragmatic, multi-centre randomised controlled trial evaluating the clinical and cost effectiveness of adding an optimised exercise programme for hands and upper limbs to best practice usual care for patients with RA.

**Methods/design:**

480 participants with problematic RA hands will be recruited through 17 NHS trusts. Treatments will be provided by physiotherapists and occupational therapists. Participants will be individually randomised to receive either best practice usual care (joint protection advice, general exercise advice, functional splinting and assistive devices) or best practice usual care supplemented with an individualised exercise programme of strengthening and stretching exercises. The study assessors will be blinded to treatment allocation and will follow participants up at four and 12 months. The primary outcome measure is the Hand function subscale of the Michigan Hand Outcome Questionnaire, and secondary outcomes include hand and wrist impairment measures, quality of life, and resource use. Economic and qualitative studies will also be carried out in parallel.

**Discussion:**

This paper describes the design and development of a trial protocol of a complex intervention study based in therapy out-patient departments. The findings will provide evidence to support or refute the use of an optimised exercise programme for RA of the hand in addition to best practice usual care.

**Trial registration:**

Current Controlled Trials ISRCTN89936343

## Background

Rheumatoid arthritis (RA) is the most common inflammatory polyarthritis [1]. It is a chronic unpredictable disorder that can cause persistent joint pain, joint damage and long-term disability (especially in the hands and feet). The prevalence of RA is 1.16% in women and 0.44% in men, increasing with age to 5% in those aged over 55 [1]. Five years after diagnosis, 40% of people with RA have relatively normal function (13% in remission), 44% have mild to moderate disability, and 16% have marked functional disability [2]. Particular problems for the hands and wrists are inflammation, deformity, pain, weakness and restricted mobility resulting in loss of function [3].

Although there is no cure for the disease, there are increasingly effective drug treatments that can reduce the impact of the disease, notably disease-modifying anti-rheumatic drugs (DMARDs). The overall goals of management are to prevent or control joint damage, maximise function and decrease pain [4]. All current UK clinical guidelines for the management of RA recommend the use of physiotherapy (PT) and occupational therapy (OT) as an adjunct to drug treatment [5,6]. The three most common components of PT/OT for RA hands are exercise therapy, joint protection advice and provision of functional splinting and assistive devices [7].

A systematic review [8] of six randomized controlled trials (RCTs) of the effectiveness of exercise programmes in RA for the whole body concluded that dynamic exercise (aerobic capacity and/or muscle strength training) was effective in improving muscular endurance and strength, without detrimental effects on disease activity or pain. The number of RCTs that have specifically investigated the effects of exercise on RA hands is limited to five small studies (n=55,100, 67, 52 and 57 patients respectively) with mostly short term follow up of a few months [9-13]. Each of these studies demonstrated small improvements in hand strength or function using exercise, with no increase in joint swelling, pain or disease activity. The long term effectiveness of exercise for RA hands has not been established.

The economic cost of RA is thought to be substantial for both the individual patient and society as a whole [4,14]. The highest costs are associated with those patients that have poor and declining function early on in their disease [15].

### Aims

This paper describes the trial protocol of a large pragmatic randomised controlled trial to evaluate the clinical and cost effectiveness of adding an optimised exercise programme for hands and upper limbs to best practice usual care for patients with RA. An additional aim is to describe, qualitatively, the experience of participants with a particular emphasis on patient expectation, exercise behaviours, and reasons for adherence/non-adherence.

## Methods

### Trial design

**Figure 1:**
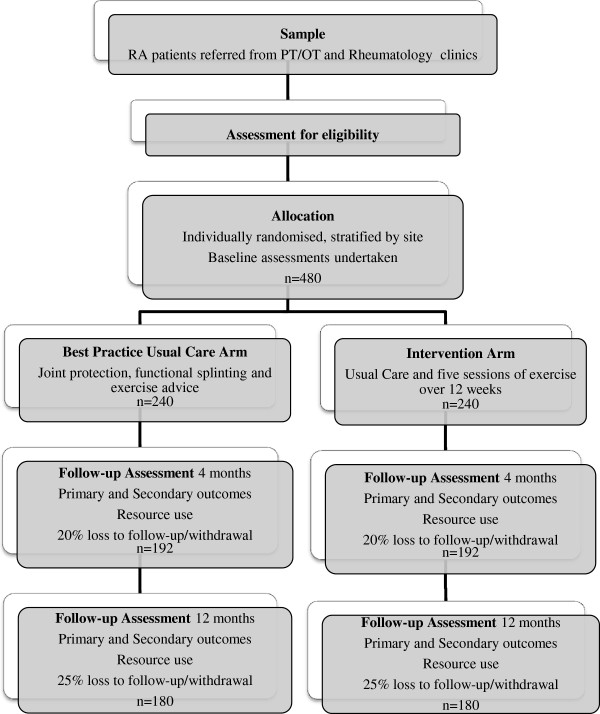
Flowchart of study design.

The SARAH trial is a pragmatic, multi-centre randomised controlled trial (Figure 1).

### Participants

480 participants will be recruited from direct referrals from Rheumatology clinics and from those referred to PT/OT clinics. 17 NHS trusts in England will recruit participants.

In addition to the recruitment of newly referred patients, a number of the Rheumatology and some of the PT/OT participating departments have a review register of ‘chronic’ patients, periodically called in for review appointments. Provided that these patients meet the selection criteria described below, they will be approached to participate in the trial.

### Selection criteria

All patients with Rheumatoid Arthritis meeting the American College of Rheumatology clinical and immunological criteria [4], with pain and dysfunction of the hands and/or wrist joints who are either not on a disease modifying medication (DMARD), or who have been on a stable DMARD regimen for three months or more, will be included in the trial. Those fulfilling any of the following criteria will be excluded:

i. Aged less than 18 years.

ii. Patients who have experienced upper limb joint surgery, or fracture, in the previous six months.

iii. Patients on a waiting list for upper limb orthopaedic surgery.

iv. Patients who are pregnant.

Patients will be asked to give written informed consent according to principles of Good Clinical Practice and the Declaration of Helsinki [16]. At the time of consent, outcome assessors will collect baseline measures.

### Randomisation

Randomisation to the exercise programme or usual care will be via a central telephone randomisation service at Warwick Clinical Trials Unit, University of Warwick. The randomisation schedule will be prepared by the trial statistician (CM). Randomisation will be stratified by centre using a variable block size.

### Allocation concealment

Eligibility checks will be performed, and consent for randomisation taken. The research clinician will then telephone the randomisation service, and only once the patient is registered in the trial, will the random allocation be generated. Hence allocation will be concealed.

### Blinding

The outcome assessor will be blind to the group allocation of the participant and will be independent of intervention delivery. Participants will be requested not to disclose group allocation to the outcome assessor. If an outcome assessor is unblinded, this will be recorded. All assessors will be asked to guess which allocation they think the participant has been given at both follow-up time points. The patients and therapists providing the treatment cannot be blinded to the group allocation.

### Baseline assessment

After participants have been assessed for eligibility and consent has been gained, baseline assessment will be carried out. Questionnaires will be completed whilst the outcome assessor conducts randomisation followed by a physical assessment (both participant and assessor will be unaware of allocation at this appointment). The baseline measures are summarised in Table 1. A Research Clinic Questionnaire will record demographic details (age, sex, date of RA diagnosis, ethnicity, marital status, hand dominance) and the Michigan Hand outcomes Questionnaire (MHQ) [17], The MHQ has shown to be a reliable, valid and responsive measure for an RA population [18-20] and contains six domains (1) overall hand function, (2) Activities of Daily Living (ADL), (3) pain, (4) work performance, (5) aesthetics, and (6) patient satisfaction. Scores range from 0 to 100, with higher scores indicating better performance, except for the pain scale. For the pain scale, a higher score indicates more pain.

**Table 1:** Summary of measures to be collected

**Domain**	**Data source**	**Measures – Instrument (Scale – high value is better score unless specified)**	**Time points**
Function	Research Clinic Questionnaire (participant reported)	Michigan Hand Outcomes Questionnaire (MHQ)– overall Hand function score (0–100)	0, 4, 12
		Michigan Hand Outcomes Questionnaire (MHQ)– overall score (0–100)	
Pain	Research Clinic Questionnaire	Pain sub-scale of MHQ (0–100; high score is worse)	0, 4, 12
		‘Troublesomeness’ rating (0–20; high score is worse)	
Impairment	Research Clinic Examination (performed by outcome assessor)	Joint deformity (MCPJ only) – goniometer (Degrees; high score is worse)	0, 4, 12
		Wrist range of motion (flexion/extension) – goniometer (Degrees)	
		Finger range of motion (combined flexion and combined extension) – Ruler (mm; high score is worse for combined flexion)	
		Thumb opposition range of motion - observation (0–10)	
		Dexterity - timed 9 hole peg test (Seconds; high score is worse)	
		Grip and Pinch Strength – dynamometer (Newtons)	
Disease Activity	Medical Records Research Clinic Examination	Erythrocyte sedimentation rate (ESR – mm/h) and/or C-Reactive protein (CRP – mg/l) blood test	0, 4, 12
		Hand and wrist joint tenderness and swelling count – examination (0–22; high score is worse)	
Health-related Quality of Life	Research Clinic Questionnaire	SF-12(0-100)	0, 4, 12
		EuroQol EQ-5D (health utility)(0–1)	
Self-efficacy	Research Clinic Questionnaire	7 item questionnaire	0, 4, 12
Satisfaction	Research Clinic Questionnaire	Treatment satisfaction item	4,12
		Satisfaction sub-scale of MHQ (0–100)	
Global Change	Research Clinic Questionnaire	Participant-rated global change question(7 point Likert scale)	4, 12
Adherence	Research Clinic Questionnaire	5 item questionnaire	0, 4, 12
Economics	Research Clinic Questionnaire	Resource use questionnaire	4, 12

The questionnaire will also contain measures of pain troublesomeness [21], self-efficacy [22], the EuroQol EQ5D [23], the 12 item short form health survey (SF-12) [24], health economics-related questions (employment status, sick days in past month due to RA in wrists/hands, benefits, highest educational qualification, household income) and treatment preference. Blood test results (CRP, ESR, serum rheumatoid factor) and current medication (prescribed and as required in last 7 days) will be taken from hospital and prescription records. The outcome assessor will be present to answer any questions regarding the measures but will be trained not to influence the participant’s responses.

Following completion of the case report form, a physical assessment will be performed in a standardised order and standardised positions. This will include the measurement of joint deformity (MCP ulnar/radial deviation in maximum pronation, where ulnar deviation is recorded as a positive value) and active range of motion (wrist flexion and extension from the neutral position with a goniometer [25], combined finger flexion according to Ellis and Bruton [26], combined finger extension and thumb opposition according to Kapandji [27]). A modified swollen and tender joint count (22 joints of hand and wrist [28]) will be taken, along with a test of upper limb dexterity (Nine-hole peg test according to Mathiowetz et al. [29]). Finally, two forms of grip strength (full-hand and tripod pinch will be measured using the MIE Digital Grip Analyser [30]. The standard test position recommended by the American Society of Hand Therapists will be used [31]. Patients will be sat in a straight-backed chair without arm rests, feet flat on floor, the shoulder of the assessed limb will be relaxed by the side, the elbow flexed to 90 degrees, the wrist will be extended and in ulnar deviation between 0 and 15 degrees and the forearm rotated to neutral pronation/supination. The mean of three maximal three-second grips will be calculated for each hand, with 60 second rests between repetitions.

### Interventions

**Figure 2:**
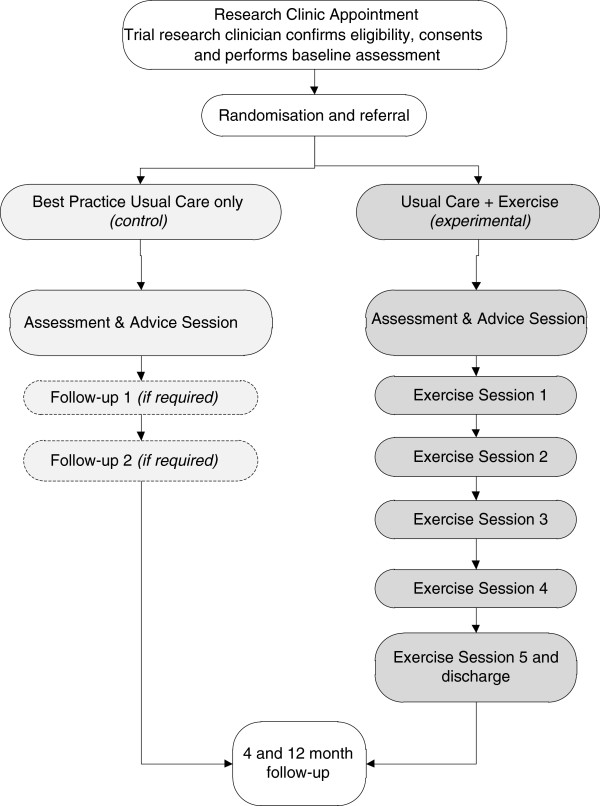
Flowchart of interventions.

The rationale and protocol for the interventions are described in a separate paper [32]. All interventions are delivered by PTs or OTs experienced in the treatment of hand and rheumatology conditions. The therapists will be independent of the recruitment and randomisation procedures and will attend a training session by the trial team and receive a training and reference manual as well as on-going support and guidance regarding the intervention to ensure quality standards are met. Therapists will be permitted to deliver both the experimental and control interventions, and each treatment session will be recorded in a treatment log. Contamination will be minimized through monitoring of treatment logs completed at each session, quality assurance visits to each therapist at the beginning of their time delivering interventions on the trial and also limitation of additional therapy materials (Patient exercise booklets, therapeutic putty, Thera-band® resistive band and hand exercisers) sufficient to only cover participants randomised to the experimental arm of the study. The aim is for all treatments to be completed within four months of the baseline assessment. Figure 2 provides an overview of the interventions.

a. Control intervention – Best Practice Usual Care only

Participants randomised to the control intervention will have between one and three sessions of outpatient therapy with a maximum total contact time of one and a half hours. Treatment will include the provision of joint protection information, splinting, assistive devices and other general advice as required. The number of sessions will depend on quantity of advice and education required and/or for review of splinting/assistive devices. This will be determined by the stage and severity of the disease. The choice of treatments was made using two principles; first, there is evidence that the treatments are effective for hand dysfunction in RA [33], and second, the treatments are consistent with current clinical guidelines [5,6]. Both control and experimental interventions were developed using focus group meetings with stake holder clinicians from across the UK.

b. Experimental intervention – Best Practice Usual Care supplemented with an optimised exercise programme

Participants randomised to the exercise programme will receive the usual care package plus an additional five sessions with a therapist over a 12 week period. The aim will be to increase hand function using exercises to stretch and strengthen the muscles and tendons, as well as mobilise the joints of the hand and wrist and improve dexterity. This will be supported by a Home Exercise Programme (reinforced by a behavioural activation approach and exercise diary) to be performed daily. This number of contacts, spread over this period, allows maximum progression of the intensity of exercise and sufficient time for a physiological response in the neuromuscular system to significantly improve function [34]. The intervention will use a standardised protocol of progression and, if necessary, regression of exercise intensity. A modified Borg scale of perceived exertion [35] will be used to regulate the intensity of resistance exercise. The programme is based on the existing evidence base, a professional consensus of UK PT/OTs and a programme that has some evidence of short-term effectiveness [36]. Adherence with the exercise programme is vital to ensure the dosage required to strengthen muscle and improve mobility is achieved. Evidence based strategies to maximise adherence will be incorporated into each treatment session, including effective goal setting and action planning [37,38].

Treatment fidelity will be evaluated by recording content of sessions on treatment logs and the number of sessions attended by participants in both arms of the trial. Quality assurance checks will be made by the clinical research fellow who will observe treatment sessions for all therapists. We will also evaluate whether participants have progressed exercises within the exercise arm using information from the treatment logs and exercise diaries.

### Other treatments

Participants may seek other forms of treatment during the follow-up period. Additional treatments, including contacts with their GP or other health professional, changes in types of medication, use of physical treatments or alternative therapies, will be recorded as a treatment outcome.

### Follow-up data collection

Follow-up data collection will be by face-to-face clinical assessment at four and 12 months. Where face-to-face clinic assessment is not possible, postal and telephone data collection methods will be used to obtain core data. The outcome measures have been described in the baseline assessment section and are detailed in Table 1. The primary outcome measure will be the 12 month Michigan Hand Outcome Questionnaire (MHQ) overall hand function subscale score.

### Sample size

A standardised mean effect size of 0.3 is reported to represent a clinically important difference in hand function in this group [39]. A previous small study using a similar intervention found a mean benefit of 0.7 in the AIMS2 with a standard deviation of 1.81 and a standardised effect size of 0.39 [36]. This suggests that in this larger, more pragmatic multi-centre trial, a standardised effect size of 0.3 in a similar function score of the MHQ is realistic and meaningful. To show this difference with 80% power at the 5% significance level, we require data on at least 352 participants (using SAS procedure GLMPOWER). Assuming a worst case scenario of 25% loss to follow-up, this would require 470 participants to be recruited initially.

Over 15 months we expect 1,200 people with hand RA to be referred to our participating centres. If half of these are assessed for study entry and 80% of these join the study, we will have 480 participants (1,200*0.5*0.8). This is our target sample size (Figure 1). The assumptions underlying the sample size calculation will be monitored by the Data Monitoring Committee.

### Statistical analysis

Patients will be analysed in the treatment group to which they were randomised, regardless of the treatment that they actually received [40]. The trial will be reported to CONSORT guidance and standards [41], and all estimates will be reported 95% confidence intervals. Demographic, clinical characteristics and baseline measurements will be presented to compare the trial groups and generalisability to clinical settings.

#### Primary outcome

The difference between the intervention and best practice usual care groups in mean MHQ overall hand function subscale score at 12 months will be analysed by a linear model, adjusted for the baseline score. Adjustment will be made for centre, sex and age. Further adjustment by disease activity, medication and other patient characteristics will be considered if there are imbalances between the intervention groups.

#### Secondary outcomes

Differences between groups for MHQ overall hand function score at 4 months, overall MHQ score at 4 and 12 months, measures of pain (sub-scale of MHQ and ‘troublesomeness’), impairment measures, self-efficacy, joint counts, and quality of life (SF-12 and EQ-5D) scores will be analysed in a similar manner to the primary outcome measure.

Analyses will also take account of baseline drug regimens (no DMARD, single DMARD, combination DMARD or biologic DMARD) and disease duration (< or > 5 years since diagnosis). The impact of therapist effects will be explored by including a random therapist effect nested within centre in the various models. The effect of missing data will be investigated using multiple imputation analysis.

Complier-average causal effect (CACE) will be estimated using an instrumental variable method [42]. Statistical analyses will be performed using SAS V9.2 software.

### Economic evaluation

The economic analysis will include a ‘within-trial’ cost-utility analysis, and modelling to estimate longer-term costs and health effects if justified by the clinical results.

For the within-trial analysis, costs incurred and Quality-Adjusted Life Years (QALYs) attained over the twelve month study period will be estimated at the individual patient level. The EuroQol (EQ-5D) will be used to generate individuals’ utility scores at 0, 4 and 12 months (using the UK Social Tariff [43]), and QALYs will then be calculated over this period using an area-under-the-curve approach. Similarly, self-reported resource use data collected at 4 and 12 months will be used to estimate individuals’ hand RA related health and social care costs over the 12 month study period. The cost of the exercise intervention will be estimated from PT and OT records of time spent with patients, and of any consumables used. Costs will be estimated from an NHS and Personal Social Service perspective (as recommended by NICE [44]), as well as from a broader societal perspective (including patient expenditure). Unit costs for publicly funded services will be taken from standard national sources [45], costs of private services will be obtained directly from patients. As for clinical outcomes, missing resource use and QALY data will be estimated by multiple imputation.

A regression approach will then be used to estimate between-group differences in mean costs and QALYs, adjusting for centre, sex, age and other baseline differences between the study groups. An Incremental Cost-Effectiveness Ratio (ICER), the additional cost per additional QALY gained with the exercise programme compared with best usual practice alone, will be calculated if appropriate. Interaction terms will also be used to investigate possible treatment moderators and hence to identify patient subgroups for whom treatment cost-effectiveness is predictably different: age, sex, disease activity, medication group, duration of disease, or other relevant patient characteristics.

The twelve-month time horizon of the within-trial analysis is quite limited. It is possible that extrapolation of costs and QALYs beyond this time might lead to different conclusions about cost-effectiveness. For example, if participants randomised to the exercise programme had greater quality of life at twelve months than those randomised to usual care alone, but that this health gain came at a relatively high cost, then it is possible that further accumulation of quality of life gains and/or cost savings after the first year could bring the ICER below the £20,000 to £30,000 per QALY gained threshold that is usually considered to be cost-effective in the UK [46]. If so, we will use decision-analytic modelling with probabilistic sensitivity analysis to extrapolate expected costs and health effects.

### Qualitative study

The qualitative study will help to provide a picture of issues facing patients with hand problems as a result of RA who participate in the experimental intervention arm of the study. Semi-structured interviews will be conducted by a researcher experienced in the design, collection and analysis of qualitative data. Specific topics covered will be living with RA, exercising with RA (including adherence issues), and participant experience of the SARAH trial.

We will interview enough participants to ensure we are confident that theoretical sufficiency will be achieved [47]. Therefore, we are aiming to interview up to 20 participants randomised to receive the exercise intervention. Sampling will be purposive to enable recruitment of patients who report benefitting and not benefitting from the exercise programme.

Interviews will be conducted following four and 12 month follow-up appointments either in hospitals or patient homes. The development of the interview schedule will be iterative and the questions asked may develop and change as the interviews are conducted and analysed [48].

All interviews will be recorded and then transcribed for analysis. Interpretative Phenomenological Analysis (IPA) will be used [49]. A feature of IPA is that the first steps of analysis begin early in the research process with initial data coding and is simultaneous to data collection. Initial analysis of each interview will be carried out as soon after its completion as possible following the guidelines set out by Smith et al. [50].

One in five of the interviews will be coded independently by a 2^nd^ member of the research team with experience of qualitative research (FT) to provide additional knowledge by giving a different perspective on the coding [51]. The research team will discuss the development of themes as the research progresses, once again, with the aim of providing a different perspective and enhancing the development of themes. Participants will be given a pseudonym to be used in any reports related to the study, with the option to choose their own pseudonym.

### Ethics committee approval

The SARAH trial was approved by the Oxford C Multicentre Research Ethics (MREC 08/H0606/47) Committee and by the Research and Development department of each participating centre.

## Discussion

Impairment and dysfunction of hands and upper limbs are key concerns for patients with RA and optimal conservative management has yet to be defined and documented. This pragmatic randomised controlled trial investigates whether there is additional benefit of an optimised exercise programme for hands and upper limbs when added to best practice usual care for this group of patients.

A strength of this study is that the exercise programme will be individually tailored to each patient, reflecting clinical practice, and is designed to ensure a sufficient dose of exercise is delivered. Strategies to maximise adherence to the exercise programme are an addition to an already promising intervention. The intervention is designed to fit with the usual constraints of NHS provision.

There is also methodological rigour in the design, range of validated outcome measures and mixed methods utilised that will help to understand underlying mechanisms and key patient perspectives. Long term follow-up and the large sample size will allow for a comprehensive clinical effectiveness analysis. The parallel health economics analysis will enable evaluation of cost-effectiveness of treatments which are particularly pertinent in the current economic climate.

## Competing interests

The authors declare that they have no competing interests.

## Authors’ contributions

All authors critically revised the manuscript for important intellectual content and approved the final manuscript. JA participated in the conception, design and conduct of the study, obtaining funding and will participate in interpretation of data. CB developed the behavioural activation sections of the intervention and participated in obtaining funding. SD co-ordinated the administration of the study and acquisition of data, administrative and material support. PH participated in design and conduct of the study, intervention development and will participate in interpretation of data. SEL is chief investigator, participated in conception and design of the study, initial drafting of manuscript, obtaining funding, and will participate in analysis and interpretation of data. JL participated in the design of the study, drafting of the health economics section of the manuscript and will oversee the health economics analysis. CM participated in the design of the study, obtaining funding, drafting of the statistical analysis section of the manuscript and will conduct the statistical analyses. VN participated in the design and data collection for the qualitative study, drafting of the qualitative section of the manuscript and will conduct the qualitative study analysis. FT participated in the design and analysis of the qualitative study. MRU participated in the conception, design and conduct of the study, obtaining funding, supervision and will participate in interpretation of data. MAW participated in the design and conduct of the study, lead drafting of the manuscript, and will participate in analysis and interpretation of data. EMW led the design and data collection for the qualitative study and will conduct the qualitative study analysis.

## Pre-publication history

The pre-publication history for this paper can be accessed here:

http://www.biomedcentral.com/1471-2474/13/230/prepub
